# Predictive value of motor-evoked potentials for motor recovery in patients with hemiparesis secondary to acute ischemic stroke

**DOI:** 10.1080/07853890.2023.2225144

**Published:** 2023-06-22

**Authors:** Cheng-Chang Yen, Hsin-Hung Chen, Chao-Hsien Lee, Ching-Huang Lin

**Affiliations:** a Division of Neurology, Department of Internal Medicine, Kaohsiung Veterans General Hospital, Kaohsiung, Taiwan; b Department of Medical Education and Research, Kaohsiung Veterans General Hospital, Kaohsiung, Taiwan; c Department of Health Business Administration, Meiho University, Pingtung, Taiwan

**Keywords:** Stroke, motor evoked potential, paretic limb, motor recovery

## Abstract

**Background:**

Motor recovery following a stroke is related to the initial stroke severity and corticospinal tract integrity. One of the outcomes representing corticospinal tract integrity is the motor evoked potential (MEP). This study aimed to investigate the predictive value of MEP for motor recovery in patients with acute ischemic stroke.

**Patients and Methods:**

Patients with hemiparesis secondary to initial acute ischemic stroke were enrolled. MEPs of the upper limb were assessed as preserved (MEP+) or absent (MEP-) response ≤10 days post-stroke. Fugl-Meyer assessment (FMA) was performed at baseline and post-stroke at 30 and 90 days. A modified Rankin scale (mRS) was conducted at 90 days post-stroke. Patients were divided into two groups according to the highest FMA score of MEP- patients. Generalized estimating equations and logistic regression were used for our study analysis.

**Results:**

Sixty-one participants were included in this study. The highest FMA score of MEP- patients ≤10 days after stroke was 38. Among patients with an initial FMA score ≤38, FMA scores at 30 and 90 days post-stroke were significantly higher in MEP + patients than in MEP- patients. Proportional recovery at 30 and 90 days post-stroke was significantly higher in MEP + patients than in MEP- patients. MEP + patients had a higher percentage of good functional outcomes than MEP- patients, without statistical difference. Among patients with initial FMA score >38, FMA scores were 60.4 ± 4.8 and 63.9 ± 2.9 and proportional recovery was 65.2 ± 27.0% and 83.7 ± 24.6% at 30 and 90 days post-stroke, respectively.

**Conclusions:**

Among patients with moderate-to-severe ischemic stroke, MEP + patients had better motor recoveries (approximately 70%) than MEP– patients at 90 days post-stroke. MEP + patients had better functional outcomes than MEP- patients.

## Summary

Among patients with moderate-to-severe ischemic stroke, those with positive motor-evoked potentials (MEPs) had better motor recovery than those with negative MEPs at 90 days post-stroke.

Assessment of motor-evoked potentials is a reliable method for predicting motor recovery in patients with moderate-to-severe ischemic stroke.

Corticospinal tract function of patients with acute ischemic stroke was tested with transcranial magnetic stimulation.

## Introduction

Stroke is one of the major burdens of medical care and the second leading cause of death worldwide [1]. Stroke survivors often face various disability problems; for example, limb movement dysfunction is one of the most common sequelae, which greatly affects patients’ daily life. In addition, hemiplegia or hemiparesis are major symptoms that substantially affect the daily life functions of patients with stroke. In Taiwan, stroke is the fourth leading cause of mortality and intensive post-stroke rehabilitation has been provided by National Health Insurance Administration to improve patient’s daily function of patients with stroke. Therefore, motor recovery after a stroke is an important issue and should be investigated widely [2,3].

Transcranial magnetic stimulation (TMS), first proposed in 1985, is one of the tools used to measure motor-evoked potentials (MEPs) [4]. MEP is generally believed to reflect the integrity of the corticospinal tract and is related to the excitement of the entire motor system [5]. Many studies [6–10] have shown that MEPs are a sensitive predictor of good functional recovery. However, a study by Arac et al. revealed contrary results [11]. A systematic review [6] concluded that the upper limb impairment level and function at baseline and intact MEPs showed consistently strong associations with upper limb recovery following stroke. However, the assessment scales used to assess the prognosis of stroke in the above studies are varied and include Fugl-Meyer assessment (FMA), Medical Research Council scale, Canadian Neurological Scale, Barthel Index, and The Action Research Arm Test.

The Fugl-Meyer assessment (FMA) is a reliable, validated measure of extremity impairment and has been used to assess motor recovery after stroke in many studies [8,12–14]. In 2008, Prabhakaran et al. [14] described that motor recovery, assessed with the upper extremity Fugl-Meyer motor score after stroke, had a proportional relationship with initial impairment (recovery ≅ 0.70 × initial impairment). Many studies have concluded that the integrity of the corticospinal tract is one of the most important factors in stroke outcome or proportional recovery [9,12,14,15]. Then, Byblow et al. [12,16] found that motor recovery in the upper limbs 3–6 months after a stroke had a proportional relationship with initial impairment among patients with stroke who preserved MEP, indicating an intact corticospinal tract. However, the proportional relationship between initial impairment and recovery over time has been criticized in some articles [17–19]. Hawe et al. [18] demonstrated that proportional recovery is highly biased because of mathematical coupling and the impact of the ceiling effect in FMA.

Both initial motor impairment and the integrity of the corticospinal tract that can be detected with MEP affect stroke recovery and outcome. In addition, there are few studies on the predictive value of MEP in patients with different stroke severity. The results of studies on proportional motor recovery of patients with ischemic stroke have also been questioned. We attempted to use the initial motor impairment, FMA score, to group the participants in this study, and then performed multiple measurements at different times within 3 months after acute ischemic stroke to analyze the initial motor function and subsequent recovery. This study aimed to assess the predictive value of MEP and proportional motor recovery in patients with ischemic stroke of varying initial severity.

## Participants and methods

### Participants

**Figure 1.**
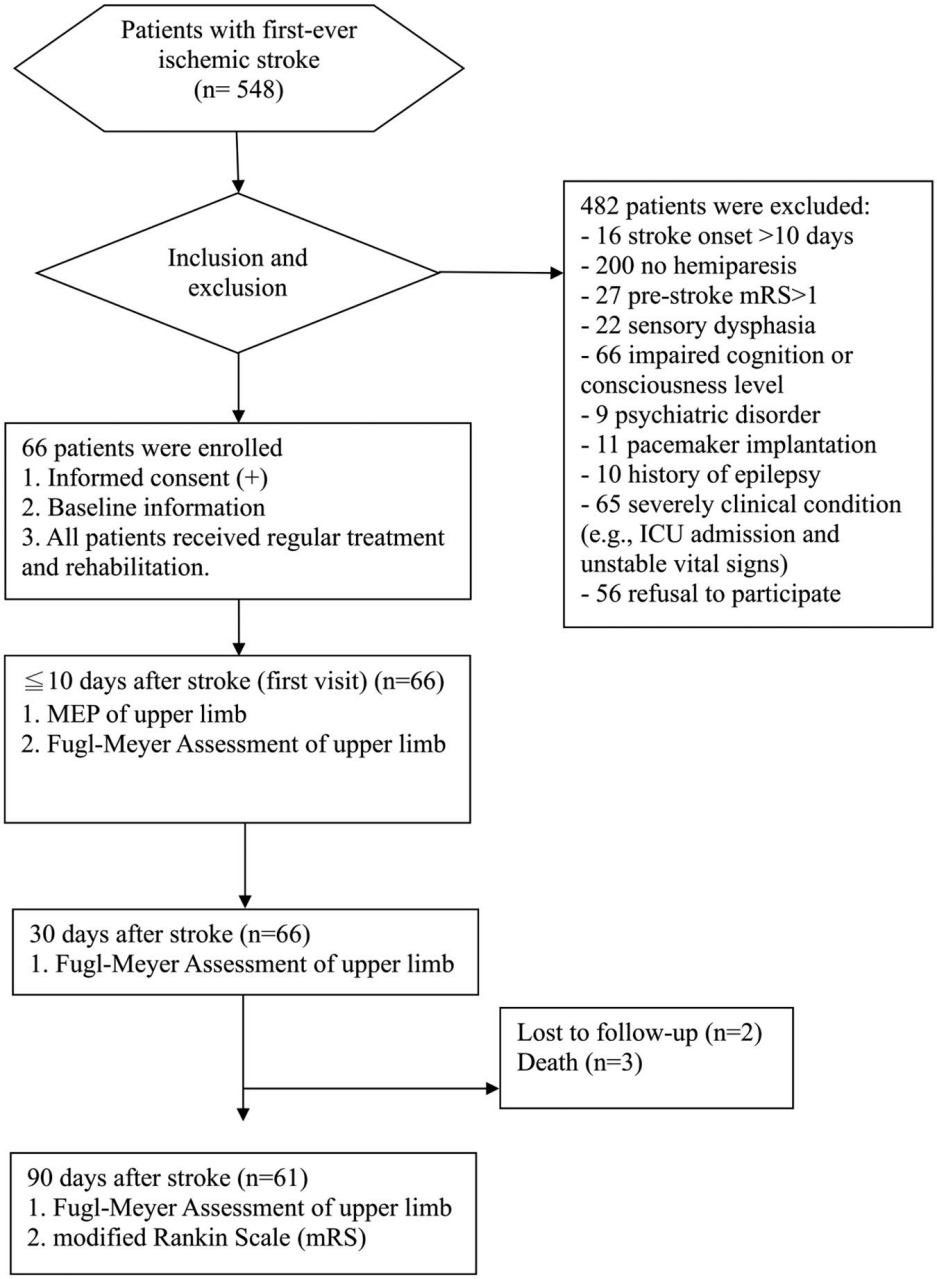
Study flow chart. MEP: motor-evoked potential; ICU: intensive care unit.

All patients with acute ischemic stroke who were admitted to our institution between January 2018 and July 2019 were enrolled in this prospective study (Figure 1). Patients were included in the analysis only if they met all the following criteria: (1) first-ever ischemic stroke; (2) representation of hemiparesis or hemiplegia; (3) stroke onset within 10 days; (4) ≥18 years of age; (5) no impairment of cognition or consciousness. The exclusion criteria were as follows (1) pre-stroke modified Rankin scale (mRS) score of >1; (2) sensory dysphasia, dementia, or impaired cognitive function; (3) history of psychiatric disorders or suicide attempt; (4) metal implant within the head (e.g. metal clip, cochlear implant); (5) pacemaker implantation; (6) history of epilepsy; and (7) pregnancy.

### Standard protocol approval and patient consent

This study was approved by the ethics committee of our institution (approval number: VGHKS17-CT8-16). Written informed consent was obtained from all patients or their next of kin.

### Clinical assessment

Ischemic stroke was diagnosed by a neurologist based on clinical manifestations and either brain computed tomography (CT) or magnetic resonance imaging (MRI). All patients with acute ischemic stroke received routine rehabilitation and treatment according to the treatment guidelines of the Taiwan Stroke Society for acute ischemic stroke. We collected the patients’ baseline information, including age, sex, smoking habit, drinking habit, body weight, height, comorbidities, and the National Institutes of Health Stroke Scale (NIHSS) score. NIHSS was assessed by a neurologist. Stroke location was determined using a brain CT scan and/or MRI and was categorized as cortical, subcortical, or brain stem. Motor function of the upper extremities was measured with the FMA (range: 0–66) at ≤10 days and 30 and 90 days after stroke onset by another neurologist. Proportional recovery was calculated as follows: Proportional recovery = (subsequent FMA score − initial FMA score)/(66 − initial FMA score). The subsequent FMA score was the score at 30 days and 90 days after the stroke. Post-stroke functional outcome was evaluated using the mRS at 90 days after stroke onset by the same neurologist. An mRS score of ≤1 was defined as a good functional outcome.

### Transcranial magnetic stimulation

TMS sessions were conducted by a trained medical examiner on the same day of the clinical assessment at ≤10 days after stroke onset. The paretic upper extremity was examined. The TMS session was conducted according to the practical guide published by the International Federation of Clinical Neurophysiology [20,21]. We employed monophasic electromagnetic stimulators (Magstim 200 Mono Pulse, MAGSTIM Co., UK). Patients were examined while lying on a bed with their heads slightly protruding beyond the edge of the bed in a quiet room. MEPs were recorded with surface electrodes from the first dorsal interosseous muscle of the upper extremity. In the TMS session of the upper extremity, we used a figure-of-eight coil. The juncture of the two wings of the coil was placed approximately 5 cm lateral to and 0–1 cm anterior to the vertex and contralateral to the paretic extremity, where the largest MEP in the paretic first dorsal interosseous muscle was elicited.

TMS was applied while patients contracted the paretic target muscle slightly. Preserved MEP (MEP+) was considered if MEPs with peak-to-peak amplitude of ≥200 µV were induced by TMS with a stimulation intensity of up to 100%. If no voluntary movement could be produced in the paretic target muscle, we requested the patient to contract it with effort. Then, preserved MEP (MEP+) was also considered if MEPs of any amplitude with a consistent latency were observed in at least 50% of the trials. On the other hand, a nonpreserved MEP (MEP-) meant that no MEP was obtained after 100% intensity stimulation of TMS while the patient was attempting to contract the paretic target muscle with effort. We classified our patients as MEP + or MEP- based on the presence of MEPs in the paretic upper extremity.

### Data analysis

We first showed the clinical characteristics of the study participants. All continuous variables are presented as mean ± standard deviations and ranges (minimum, and maximum values). All categorical variables are presented as numbers and percentages. We divided all participants into two groups based on initial FMA and MEP, as follows: Group A, patients with initial FMA scores less than or equal to the highest FMA score for MEP- patients; Group B, patients with initial FMA scores higher than the highest FMA score for MEP- patients. For univariate analysis of clinical characteristics between the MEP- and MEP + patients in Group A, we used Student’s *t*-tests for continuous variables and the chi-square tests (or Fisher’s exact tests) for categorical variables. Generalized estimating equations (GEEs) were used to analyze repeated measures of the FMA score between MEP + and MEP- patients in Group A. Working correlation matrix was autoregressive. Linear scale response was applied for the type of model. If the interaction between time and MEP groups showed statistical significance, analysis of the simple main effect was performed. Logistic regression was applied for the analysis and comparison of functional outcome (mRS) at 90 days after stroke between MEP + and MEP- patients in Group A. All statistical analyses were performed using the SPSS software version 22.0 (IBM Corp., Armonk, NY, USA). The threshold for statistical significance was set at *p* < 0.05.

## Results

Sixty-six patients with acute first-ever ischemic stroke were initially enrolled between January 2018 and July 2019. Of these, two patients were lost to follow-up and three patients died. Ultimately, 61 patients (mean age, 66.2 ± 9.4 years; sex, 32 men and 29 women) were included in the analysis. The baseline characteristics of all patients are presented in Table 1. The average time interval between the day of the first visit and stroke onset was 6.7 ± 1.8 days. The average NIHSS score was 6.9 ± 3.7. The average FMA score was 25.9 ± 18.7. MEP + patients had a higher FMA score than MEP- patients (35.9 ± 18.2 and 13.2 ± 9.2, respectively). The range of the initial FMA score in MEP- patients was 4–38. The highest FMA score among MEP- patients was 38. All patients with stroke with initial FMA scores of >38 were MEP+. Therefore, Group A and Group B comprised patients with initial FMA scores of ≤38 and >38, respectively.

**Table 1.** Clinical characteristics of all patients with stroke.

	Total (*n* = 61)	Range
Age (years)	66.2 (9.4)	(43–84)
Sex (male)	32 (52.5%)	
First visit day^a^	6.7 (1.8)	(2–10)
Smoking	21 (34.4%)	
Drinking	7 (11.5%)	
BMI (kg/m^2^)	25.6 (3.7)	(17.2–33.4)
Comorbidity		
Hypertension	50 (82.0%)	
Diabetic mellitus	26 (42.6%)	
Transient ischemic attack	1 (1.6%)	
Dyslipidemia	46 (75.4%)	
Atrial fibrillation	7 (11.5%)	
Myocardial infarction	1 (1.6%)	
Chronic renal disease	3 (4.9%)	
Chronic liver disease	2 (3.3%)	
COPD	4 (6.6%)	
Stroke Location		
Cortex	10 (16.4%)	
Subcortex	34 (55.7%)	
Brain stem	17 (27.9%)	
TOAST classification		
Large artery	11 (18.0%)	
Small vessel	16 (26.2%)	
Cardioembolism	6 (9.8%)	
Cryptogenic	28 (45.9%)	
NIH Stroke Scale (NIHSS)	6.9 (3.7)	(2–18)
Fugl-Meyer assessment score	25.9 (18.7)	(4–65)
MEP+	35.9 (18.2)	(8–65)
MEP−	13.2 (9.2)	(4–38)

Category variable: n (percentage), Continuous variable: mean (standard deviation) and range.

^a^ Time interval (days) between stroke onset and first visit.

COPD: chronic obstructive pulmonary disease; NIH: National Institutes of Health; BMI: body mass index; MEP: motor-evoked potential.

**Figure 2.**
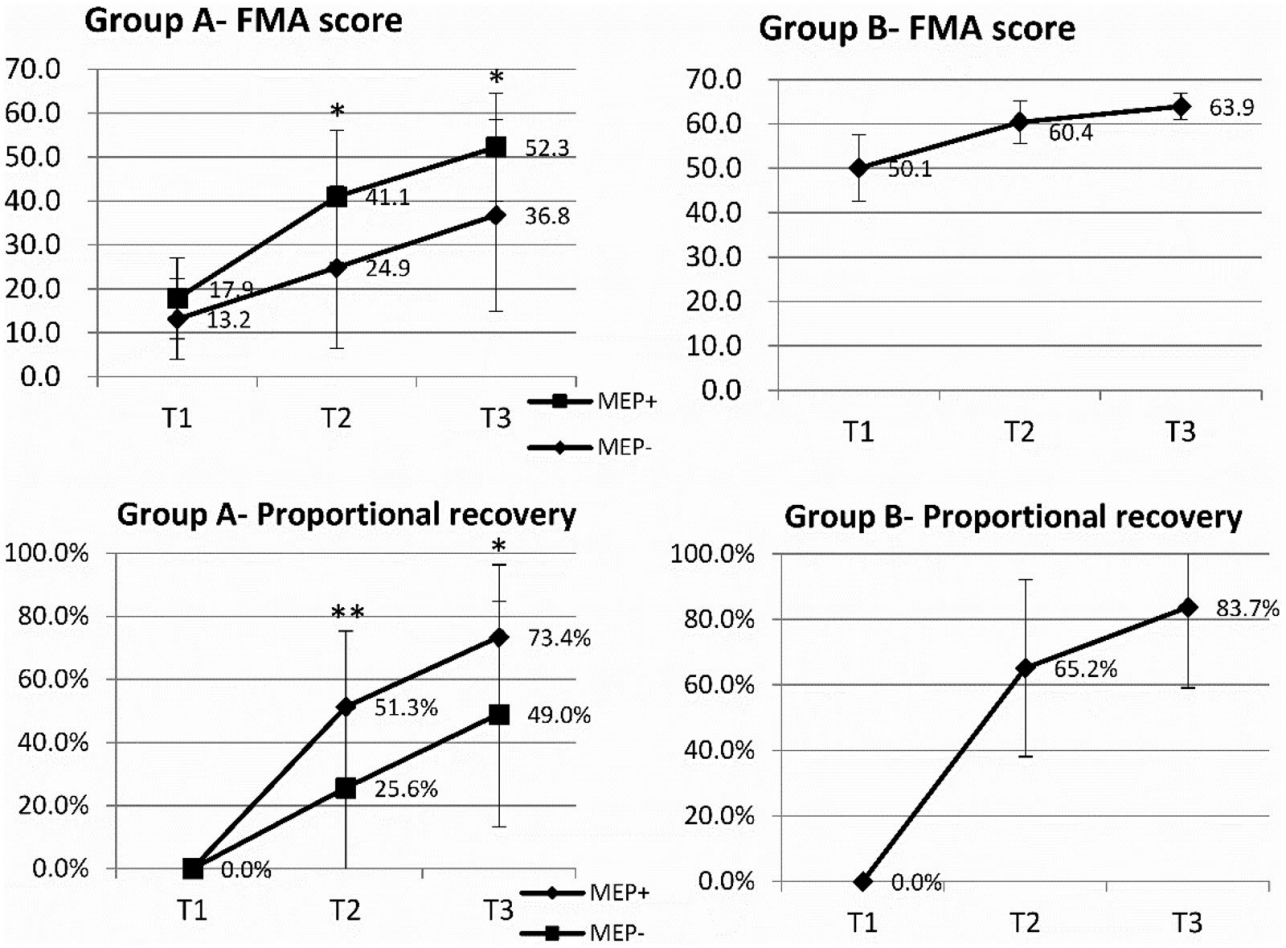
Fugl-Meyer Assessment (FMA) score and proportional recovery over time in the two groups. T1: ≤10 day after stroke, T2: 30-day after stroke, T3: 90-day after stroke. *p < 0.05, **p < 0.001

The clinical characteristics of patients with patients in Groups A and B are presented in Table 2. Within Group A, MEP + patients were significantly older than MEP- patients (age, 71.1 ± 8.2 and 65.0 ± 9.3 years, respectively). There was no statistical difference in sex, comorbidity, stroke location, and the trial of ORG 10172 in acute stroke treatment classification (TOAST classification) between MEP + and MEP- patients in Group A. The NIHSS score for MEP + and MEP- patients in Group A was 7.0 ± 2.6 and 8.8 ± 4.1, respectively, and no statistical difference was observed. The initial FMA scores for MEP + and MEP- patients in Group A were 17.9 ± 9.2 and 13.2 ± 9.2, respectively; no statistical difference was observed. Among patients in Group B, the average age, NIHSS score, and initial FMA score were 64.0 ± 9.7 years, 4.3 ± 1.6, and 50.1 ± 7.5, respectively. Motor recovery after stroke is presented in Table 3. In Group A, motor function, including FMA scores and proportional recovery, improved over time in both MEP- and MEP + patients. In group B, a similar improvement in motor function was found (Table 3). Changes in the FMA score and proportional recovery over time are illustrated in Figure 2.

**Table 2.** Clinical characteristics of patients with stroke with an initial FMA score of ≤38 (group A) or >38 (group B).

	Group A (*n* = 42)			Group B (*n* = 19)
	MEP+ (*n* = 15)	MEP– (*n* = 27)	*p*	
Age (years)	71.1 (8.2)	65.0 (9.3)	.040	64.0 (9.7)
Sex (male)	8 (53.3%)	15 (55.6%)	.890	9 (47.4%)
Smoking	5 (33.3%)	10 (37.0%)	.810	6 (31.6%)
Drinking	0 (0.0%)	4 (14.8%)	.279^a^	3 (15.8%)
BMI (kg/m^2^)	25.2 (2.3)	25.3 (3.8)	.934	26.2 (4.6)
Comorbidity				
Hypertension	11 (73.3%)	21 (77.8%)	1.00^a^	18 (94.7%)
Diabetic mellitus	5 (33.3%)	13 (48.1%)	.353	8 (42.1%)
Dyslipidemia	10 (66.7%)	21 (77.8%)	.481^a^	15 (78.9%)
Atrial fibrillation	2 (13.3%)	2 (7.4%)	.608^a^	3 (15.8%)
Myocardial infarction	1 (6.7%)	0 (0.0%)	.357^a^	0 (0.0%)
Chronic renal disease	0 (0.0%)	0 (0.0%)		3 (15.8%)
Chronic liver disease	0 (0.0%)	1 (3.7%)	1.00^a^	1 (5.3%)
COPD	3 (20.0%)	1 (3.7%)	.122^a^	0 (0.0%)
Stroke location			.558	
Cortex	1 (6.7%)	5 (18.5%)		4 (21.1%)
Subcortex	9 (60.0%)	15 (55.6%)		10 (52.6%)
Brain stem	5 (33.3%)	7 (25.9%)		5 (26.3%)
TOAST classification			.260	
Large artery	4 (26.7%)	2 (7.4%)		5 (26.3%)
Small vessel	4 (26.7%)	5 (18.5%)		7 (36.8%)
Cardioembolism	1 (6.7%)	2 (7.4%)		3 (15.8%)
Cryptogenic	6 (40.0%)	18 (66.7%)		4 (21.1%)
NIH Stroke Scale (NIHSS)	7.0 (2.6)	8.8 (4.1)	.140	4.3 (1.6)
Initial FMA score	17.9 (9.2)	13.2 (9.2)	.122	50.1 (7.5)

Category variable: *n* (percentage), Continuous variable: mean (standard deviation).

Student’s t-tests for comparison of continuous variables and chi-square tests (or Fisher’s exact tests) for comparison of categorical variables between MEP + and MEP- patients in group A.

COPD: chronic obstructive pulmonary disease; FMA: Fugl-Meyer assessment; BMI: body mass index, NIH: National Institutes of Health.

^a^ Fisher’s exact test.

**Table 3.** Motor recovery after a stroke at different time points.

	Group A		Group B
Post-stroke	MEP (+)	MEP (–)	MEP (+)
FMA score			
** **≤10 day	17.9 (9.2)	13.2 (9.2)	50.1 (7.5)
** **30 days	41.1 (15.1)	24.9 (18.4)	60.4 (4.8)
** **90 days	52.3 (12.3)	36.8 (21.9)	63.9 (2.9)
Proportional recovery (%)			
** **≤10 days	0	0	0
** **30 days	51.3 (24.1)	25.6 (26.5)	65.2 (27.0)
** **90 days	73.4 (22.9)	49.0 (35.8)	83.7 (24.6)

Mean (standard deviation).

MEP: motor-evoked potential; FMA: Fugl-Meyer assessment.

GEE analysis of motor recovery in patients with ischemic stroke in Group A is presented in Table 4 (Supplemental Material, Table S1, and Table S2). The difference in the FMA score and proportional recovery at 30 days and 90 days after stroke was significant between the MEP + and MEP- patients. Simple main effects of MEP and time were analyzed. The FMA scores of MEP + patients were significantly higher than those of MEP- patients at 30 (Wald X^2^ 9.13, *p* < 0.05) and 90 days (Wald X^2^ 4.09, *p* < 0.05) after stroke. Proportional recovery in MEP + patients was significantly higher than those of MEP- patients at 30 days (Wald X^2^ 13.90, *p* < 0.001) and 90 days after stroke (Wald X^2^ 7.84, *p* < 0.05). Regardless of MEP + or MEP- patients, the FMA score and proportional recovery became significantly higher over time.

**Table 4.** Comparison of FMA score and proportional recovery at different time points between MEP + and MEP- patients in group A.

	T1	T2	T3	Wald X^2,a^	Post-hoc test
FMA score					
MEP (+)	17.9 (9.2)	41.1 (15.1)	52.3 (12.3)	175.72**	T1 < T2,T1 < T3,T2 < T3
MEP (−)	13.2 (9.2)	24.9 (18.4)	36.8 (21.9)	61.26**	T1 < T2,T1 < T3,T2 < T3
Wald X^2,b^		9.13*	4.09*		
Proportional recovery (%)					
MEP (+)	0	51.3 (24.1)	73.4 (22.9)	209.11**	T1 < T2,T1 < T3,T2 < T3
MEP (−)	0	25.6 (26.5)	49.0 (35.8)	58.57**	T1 < T2,T1 < T3,T2 < T3
Wald X^2,b^		13.90**	7.84*		

T1: ≤10 days after stroke, T2: 30 days after stroke, T3: 90 days after stroke.

^a^ GEE of simple time (T1, T2, T3) effect.

^b^ GEE of simple group (MEP+, MEP-) effect.

* *p* < 0.05, **p < 0.001.

MEP: motor-evoked potential; GEE: generalized estimating equation; FMA: Fugl-Meyer assessment.

Eight (19.0%) patients in Group A had good functional outcomes (Table 5). Ten (52.6%) patients in Group B had good functional outcomes at 90 days after stroke. Within Group A, good functional outcomes of MEP + patients were higher than that of MEP- patients (26.7% and 14.8%, respectively). However, no statistically significant difference was noted (adjusted OR = 9.08, *p* = 0.059).

**Table 5.** Analysis of good functional outcome (mRS ≤1) at 90-day after stroke.

	mRS ≤ 1	mRS > 1	Adjusted OR^a^	95% CI (lower-upper)	*p*
Group A	8 (19.0%)	34 (81.0%)			-
MEP+	4 (26.7%)	11 (73.3%)	9.08	0.92-89.95	0.059
MEP−	4 (14.8%)	23 (85.2%)	Reference	-	-
Group B	10 (52.6%)	9 (47.4%)			

^a^ Adjusted variables: age.

OR: odds ratio; mRS: modified Rankin scale; MEP: motor-evoked potential; CI: confidence interval.

## Discussion

Previous studies [7,22,23] have demonstrated that MEP is a reliable tool for predicting motor recovery as well as functional outcomes. In our study, among patients with acute ischemic stroke, those with moderate-to-severe motor impairment of the upper extremity (FMA score ≤38), MEP + patients recovered significantly better than MEP- patients at 30 and 90 days after stroke. Moreover, a good functional outcome was more frequent among MEP + patients (26.7%) than among MEP- patients (14.8%). However, among patients with acute ischemic stroke of mild-to-moderate motor impairment of the upper extremity, MEP had less predictive value because all of these patients were MEP+.

Among many prognostic factors of stroke, initial severity is one of the vital factors. Previous research included 58 studies and found that initial measures of upper limb impairment and function were significant predictors of upper limb recovery [6]. Therefore, the initial FMA score that indicated initial impairment was an important prognostic factor for patients with acute ischemia. We found significant differences in the initial severity of motor impairment among all MEP + and MEP- patients (mean initial FMA scores: 35.9 ± 18.2 and 13.2 ± 9.2, respectively). Therefore, we grouped the patients by the initial FMA score for further analysis. We divided patients into those with FMA scores of >38 and ≤38 because patients with FMA scores of >38 were all positive for MEP.

Similar to many scales, the FMA as a measurement of recovery for patients with mild motor impairment is limited by a ceiling effect [13]. Therefore, to avoid the ceiling effect, it has been suggested that FMA should be used for measuring the baseline and changes in impairment only in patients with low motor function [24,25]. Herein, we observed a ceiling effect among patients with mild-to-moderate ischemic stroke with an initial FMA score of >38. During the recovery process, the changes in the FMA score may become smaller among these patients. We separated the patients with high and low initial FMA scores for analysis, which might avoid the interference of the ceiling effect on statistical analysis.

Several studies [12,14,16,26] have performed linear regression models of the upper limb ΔFMA score with predictors including initial impairment of FMA (66 - initial FMA score) and others. The upper limb ΔFMA score was defined as the change in the upper limb FMA score between the acute stage of stroke and 3 or 6 months thereafter. They found meaningful regression coefficients (approximately 0.7) for the ΔFMA score with the predictor of initial impairment. Byblow et al. [12] and Stinear et al. [16] concluded that patients could achieve approximately 63-70% of their maximum potential recovery in the upper limb 3 months after stroke in cases of MEP+. However, previous reports have repeatedly warned about testing the relation between change and initial values using correlation or regression [27,28]. A major methodological concern is mathematical coupling, which occurs when one variable directly or indirectly contains the whole or part of another, and the two variables are then analyzed using correlation or regression. Therefore, Hawe et al. [18] Hope et al. [19] and Howard et al. [17] have critiqued the conclusions of the proportional recovery after stroke drawn by previous studies. Mathematical coupling and compression to ceiling are the major issues.

In our study, among patients with initial FMA scores of ≤38 and MEP+, the average proportional recovery, ΔFMA score/initial impairment of the FMA score, at days after stroke was 73.4%. The observed motor recovery of 73.4% at 90 days after stroke was similar to those observed in previous studies [12,14,16,26]. This proportional recovery (approximately 70%) existed only in patients with initial FMA scores of ≤38 and MEP + in our study. Among patients with an FMA score of ≤38 and MEP-, the average proportional recovery at 90 days after stroke was 44.7%. We noted a relatively large standard deviation in the FMA score and proportional recovery among MEP- patients. This means that MEP- patients have a large recovery variation, and there may be some occult factors that have the potential to influence stroke recovery. A meta-analysis [6] showed that neurophysiological assessments, including MEP, somatosensory-evoked potentials, and diffusion tensor tractography, were associated with upper limb recovery after stroke in addition to the initial measures of upper limb impairment. Thus, in addition to MEP, other neurophysiological assessments could provide additional value for predicting recovery after stroke [9,29,30]. Among patients with an initial FMA score of >38, the average proportional recovery at 90 days after stroke was 83.7%. The value of proportional recovery seems to be larger in the high than in the low initial FMA group. However, this value could not accurately reflect the actual recovery due to the ceiling effect in the Fugl-Meyer scale. Hawe et al. [18] and Howard et al. [17] pointed out that the ceiling effect could increase or inflate the estimates of proportional recovery after stroke.

This study has some limitations. First, it was performed at a single institution. Second, the total sample size was limited to 61 patients. Last, the follow-up period after the stroke was 90 days. Therefore, the results cannot be applied to all patients with ischemic stroke. Further studies should be conducted to address these limitations and predict motor or functional recovery in patients with acute ischemic stroke.

## Conclusions

Not all outcomes in patients with ischemic stroke could be predicted well by MEP. We demonstrated that MEP + patients improved faster and better than MEP- patients concerning motor function measured by FMA in those with moderate-to-severe ischemic stroke within 90 days post-stroke. MEP + patients also tended to have better functional outcomes compared with MEP- patients in those with moderate-to-severe ischemic stroke. Moreover, for those with mild-to-moderate ischemic stroke, the predictive value of MEP was lower, as they had lower initial motor severity and were all MEP+. Previous studies on proportional recovery after stroke have been criticized for the mathematical coupling and the impact of the ceiling effect in FMA. In our study, proportional motor recovery at a stroke (approximately 70%) was again presented using different research and statistical methods.

## Supplementary Material

Supplemental Material

## Data Availability

The data that support the findings of this study are available from the corresponding author, Dr. Ching-Huang Lin, upon reasonable request.
