# Supplementary material for: Predictive value of motor-evoked potentials for motor recovery in patients with hemiparesis secondary to acute ischemic stroke
Source: Ann Med. 2023 Jun 22;55(1):2225144. doi: 10.1080/07853890.2023.2225144 (PMC10288919; doi:10.1080/07853890.2023.2225144)
Supplement: Supplemental Material [file IANN_A_2225144_SM5605.docx]

**Supplementary Tables:**

**Table S1 Generalized estimation equations analysis of motor recovery (FMA score) in group A**

| **Predictor Variables** | **Estimate Parameters(B)** | **SE** | **Wald X^2^** | **p** |
| --- | --- | --- | --- | --- |
| **FMA score** |  |  |  |  |
| **Intercept** | 27.35 | 14.64 | 3.49 | .062 |
| **MEP(+)** | 6.01 | 3.26 | 3.41 | .065 |
| **MEP(-)** | 0^a^ |  |  |  |
| **Time=3** | 23.59 | 3.05 | 59.81 | .000** |
| **Time=2** | 11.70 | 2.14 | 29.90 | .000** |
| **Time=3** | 0^a^ |  |  |  |
| **MEP(+) x Time=3** | 10.81 | 4.01 | 7.27 | .007* |
| **MEP(+) x Time=2** | 11.50 | 3.18 | 13.09 | .000** |
| **MEP(+) x Time=1** | 0^a^ |  |  |  |
| **MEP(-) x Time=3** | 0^a^ |  |  |  |
| **MEP(-) x Time=2** | 0^a^ |  |  |  |
| **MEP(-) x Time=1** | 0^a^ |  |  |  |
| **Age** | -0.22 | 0.23 | 0.93 | .334 |

Time= 1(≦10 days after stroke), 2 (30-day after stroke), 3 (90-day after stroke)

^a^ Set to zero because this parameter is redundant

*P<0.05, **p<0.001

**Table S2 Generalized estimation equations analysis of motor recovery (proportional recovery) in group A**

| **Predictor Variables** | **Estimate Parameters(B)** | **SE** | **Wald X^2^** | **p** |
| --- | --- | --- | --- | --- |
| **FMA score** |  |  |  |  |
| **Intercept** | 17.48 | 19.09 | 0.84 | .360 |
| **MEP(+)** | 1.64 | 1.96 | 0.70 | .403 |
| **MEP(-)** | 0^a^ |  |  |  |
| **Time=3** | 49.0 | 6.76 | 52.49 | .000** |
| **Time=2** | 25.64 | 5.00 | 26.30 | .000** |
| **Time=3** | 0^a^ |  |  |  |
| **MEP(+) x Time=3** | 24.42 | 8.85 | 7.62 | .006* |
| **MEP(+) x Time=2** | 25.66 | 7.82 | 10.78 | .001* |
| **MEP(+) x Time=1** | 0^a^ |  |  |  |
| **MEP(-) x Time=3** | 0^a^ |  |  |  |
| **MEP(-) x Time=2** | 0^a^ |  |  |  |
| **MEP(-) x Time=1** | 0^a^ |  |  |  |
| **Age** | -0.27 | 0.30 | 0.83 | .363 |

Time= 1(≦10 days after stroke), 2 (30-day after stroke), 3 (90-day after stroke)

^a^ Set to zero because this parameter is redundant

*P<0.05, **p<0.001
